# Differential Characteristics, Mechanisms, and Clinical Strategies for Perioperative Dry Eye in Presenile Cataract and Age-Related Cataract

**DOI:** 10.3390/healthcare14131960

**Published:** 2026-07-02

**Authors:** Ruisi Huang, Yuanling Xia

**Affiliations:** Department of Ophthalmology, Guizhou Medical University, Guiyang 550004, China; ruiruisi@163.com

**Keywords:** presenile cataract, age-related cataract, dry eye, perioperative period, meibomian gland dysfunction, age-stratified management, corneal nerve, oxidative stress

## Abstract

**Background/Objectives**: With the biphasic of cataract onset age, presenile cataract (40–55 years) and age-related cataract (≥70 years) demonstrate notable differences in perioperative dry eye manifestations. This review aims to systematically compare their differential characteristics, mechanisms, and management strategies. **Methods**: An indirect evidence synthesis was conducted, encompassing 71 clinical and mechanistic studies evaluating perioperative dry eye in these two patient populations. **Results**: Presenile patients predominantly present with evaporative dry eye secondary to meibomian gland dysfunction (55–70%), persisting for 3–6 months postoperatively, especially in subgroups with metabolic disorders, female sex, diabetes with rosacea, or preoperative anxiety. In contrast, age-related patients primarily exhibit mixed-type dry eye, with a shorter recovery period (1–3 months), characterized by corneal nerve demyelination and meibomian gland fibrosis. Mechanistically, the presenile group is associated with glandular functional overload, acute inflammation, and exogenous oxidative stress, while the age-related group shows degenerative degenerative atrophy, chronic low-grade inflammation (senescence-associated secretory phenotype, SASP), and impaired nerve regeneration. **Conclusions**: Perioperative dry eye exhibits inherent differences between presenile and age-related cataract patients, requiring age-stratified and subgroup-targeted management strategies for precise ocular surface care. However, due to the absence of direct head-to-head studies, these findings are derived from indirect evidence and should be considered hypothesis-generating.

## 1. Introduction

Age-related cataract is the leading cause of blindness worldwide, traditionally affecting individuals over 60 years old. However, clinical data over the past decade indicate an increasing incidence of cataract in patients aged 40–55 years, defined as presenile cataract (or premature cataract) due to early onset and premature lens opacification [1]. Simultaneously, population aging has expanded the group of age-related cataract patients over 70 years old, resulting in a “biphasic” distribution of cataract onset.

Dry eye is the most common perioperative complication of cataract surgery, with a prevalence of 45–57% [2]. It not only impairs the accuracy of preoperative biometry and intraocular lens (IOL) power calculation but also induces postoperative visual fluctuations, glare, and decreased contrast sensitivity, significantly compromising the visual outcomes and patient satisfaction after refractive cataract surgery [3]. Although research on postoperative dry eye in age-related cataract is relatively extensive, the dry eye burden in presenile cataract patients has been largely understudied. These two patient populations exhibit distinct baseline ocular surface characteristics, differential responses to surgical trauma, and divergent recovery trajectories, with each including unique high-risk subgroups. Consequently, a uniform management strategy may result in inadequate intervention for presenile patients or overtreatment for age-related patients [4].

Current guideline-based perioperative management of dry eye disease (DED), derived from published guidelines and expert consensus statements [5,6], typically includes preoperative administration of preservative-free artificial tears for 2–4 weeks, intraoperative measures such as small-incision phacoemulsification and minimization of ultrasound energy exposure, and postoperative tapering regimens of topical corticosteroids in combination with artificial tears [2,7]. However, this generalized approach may be suboptimal for presenile cataract patients, as it does not specifically target meibomian gland dysfunction (MGD), the predominant ocular surface abnormality reported in this population, with a prevalence of 55–70% [8,9]. Standard artificial tears and corticosteroids provide limited improvement in meibomian gland function, whereas targeted therapies, including thermal pulsation/meibomian gland expression, intense pulsed light (IPL) therapy, and lipid-containing tear substitutes, are not routinely incorporated into conventional perioperative protocols [10,11]. Conversely, application of the same management paradigm to older patients may result in unnecessary treatment intensity in selected cases. Age-related cataract patients more commonly present with mixed-type or aqueous-deficient dry eye characterized by age-related reduction in lacrimal gland function and tear secretion [12]. In such patients, routine implementation of MGD-oriented interventions or prolonged use of lipid-based formulations may provide limited additional benefit while increasing treatment burden and healthcare costs. In contrast, aqueous tear supplementation and anti-inflammatory therapies, such as cyclosporine A, may adequately address the predominant pathogenic mechanisms in many cases.

Most existing studies focus on a single age group or lack age stratification, and there are no direct head-to-head studies comparing perioperative dry eye between these two patient populations. Employing an “ indirect evidence synthesis” approach, this study systematically retrieves clinical and mechanistic research on both groups, extracts key data for cross-population comparison, and establishes a three-dimensional comparative framework of “characteristics–mechanisms–strategies” for perioperative dry eye in presenile and age-related cataract patients. This approach identifies differential clinical features and core pathophysiological mechanisms and proposes an age-stratified, subgroup-specific clinical management strategy, providing a theoretical foundation for precision ocular surface care.

## 2. Materials and Methods

### 2.1. Study Design and Analytical Framework

This is a narrative review that systematically compares the differential characteristics, underlying mechanisms, and clinical management strategies for perioperative dry eye between presenile cataract (40–55 years) and age-related cataract (≥70 years). A major methodological limitation is the absence of direct head-to-head studies between these two populations; therefore, this review adopts an indirect evidence integration strategy combined with a comparative analytical framework. All conclusions are presented as hypothesis-generating, requiring validation by future prospective studies.

### 2.2. Literature Search Strategy

A systematic literature search was conducted using a Boolean logic approach across the following electronic databases: CNKI, Wanfang, VIP, PubMed, Web of Science, Cochrane Library, and Embase. The search period covered from database inception to December 2025. Chinese search terms included combinations of “presenile cataract”/“early-onset cataract”/“premature cataract” AND “age-related cataract”/“senile cataract” AND “dry eye”/“perioperative dry eye”/“meibomian gland dysfunction”/“tear film”. English search terms included “presenile cataract” OR “early-onset cataract” OR “premature cataract” AND “age-related cataract” OR “senile cataract” AND “dry eye” OR “perioperative dry eye” OR “meibomian gland dysfunction” OR “tear film”. Reference lists of included articles were manually screened. The initial search yielded 1245 records. After removing duplicates (n = 412) and screening titles/abstracts (n = 833), 98 full-text articles were assessed for eligibility. Finally, 71 studies met the inclusion criteria.

**Figure 1:**
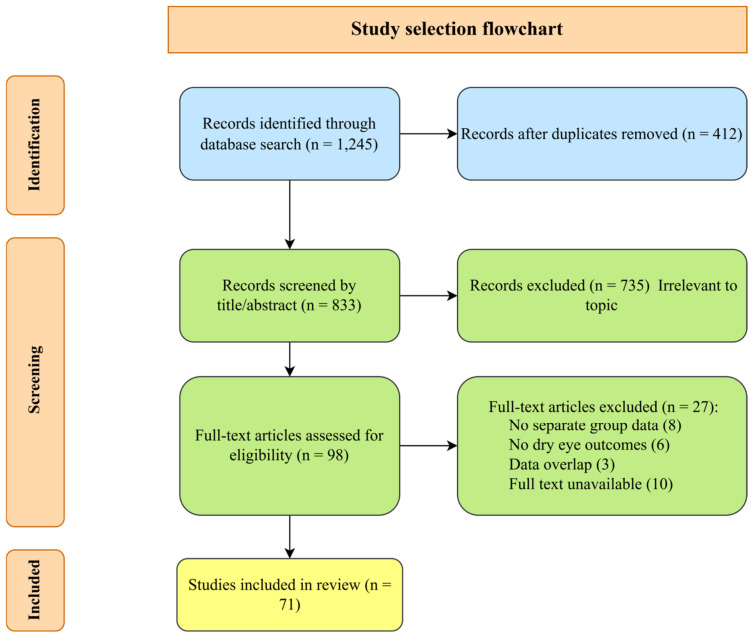
This flowchart shows the number of records identified, excluded, and finally included at each stage of screening. Exclusion reasons are listed in the corresponding boxes.

Figure 1 shows the study selection flowchart.

### 2.3. Inclusion and Exclusion Criteria

Inclusion criteria were (1) studies involving patients undergoing cataract surgery with presenile cataract (aged 40–55 years) or age-related cataract (aged ≥ 70 years); (2) studies reporting clinical characteristics, prevalence, risk factors, mechanisms, or intervention outcomes of perioperative dry eye; (3) study types including randomized controlled trials, cohort studies, case-control studies, cross-sectional studies, systematic reviews/meta-analyses, and mechanistic studies (including basic research, imaging studies, etc.); and (4) articles published in Chinese or English.

Exclusion criteria were (1) articles without full text or incomplete data; (2) duplicate publications; (3) studies exclusively on pediatric, traumatic, or secondary cataract (e.g., uveitic cataract); and (4) non-original articles (reviews, comments, case reports) without extractable data.

### 2.4. Evidence Synthesis and Analysis

Due to heterogeneity across studies in age definitions, measurement methods, and follow-up durations, a meta-analysis was not feasible. Instead, a narrative synthesis combined with comparative tables was employed. Evidence was first categorized into three dimensions: clinical characteristics, mechanisms, and management strategies. Comparative tables (as summarized in the corresponding tables) were constructed to visually present differences between the two groups. In the mechanisms section, conceptual frameworks such as “two patterns of meibomian gland dysfunction” and “two types of corneal nerve injury responses” were proposed to integrate basic and clinical evidence. All cited data were verified against original sources and annotated with reference numbers.

### 2.5. Quality Assessment and Limitation Control

Quality of included original studies was assessed using the Newcastle–Ottawa Scale (NOS) or the Cochrane risk of bias tool. For results with high heterogeneity, sensitivity analysis was performed by excluding studies with significant bias before reinterpreting the findings. This review explicitly acknowledges the current lack of head-to-head studies directly comparing perioperative dry eye between presenile and age-related cataract patients; all comparisons are based on indirect evidence integration, and the conclusions require validation by future prospective studies.

## 3. Results

### 3.1. Clinical Characteristics of Perioperative Dry Eye

#### 3.1.1. Preoperative Ocular Surface Baseline Status

1. Dry Eye Prevalence and Type Distribution

The preoperative dry eye detection rate is significantly higher in presenile cataract patients than in age-related patients. Multiple clinical observations show a dry eye prevalence of 50–65% in 40–55-year-old cataract patients, compared to 30–45% in those over 70 years old [5,13]. Age-specific differences in dry eye subtypes are evident: the presenile group is dominated by evaporative dry eye, characterized by shortened tear film break-up time (TBUT) and increased tear evaporation rate, which are closely associated with meibomian gland dysfunction (MGD) [8]. The age-related group mainly presents with mixed dry eye, combining evaporative deficiency with decreased tear secretion (lower Schirmer I test values) and mucin layer deficiency, consistent with glandular degeneration and insufficient mucin secretion [12,14].

2. Meibomian Gland Function Status

Meibomian gland dysfunction follows two age-specific patterns. Presenile cataract patients exhibit “functional overload- associated obstruction” pattern: prolonged visual display terminal (VDT) use reduces blink rate from the normal 15–20 times/min to 5–7 times/min, impairing the meibomian gland “pumping” mechanism and causing lipid stasis in ducts, while glandular structure remains intact [15]. Three-dimensional meibography shows that 67% of presenile patients exhibit a ductal dilation phenotype, with a preoperative MGD prevalence of 55–70%, mainly manifesting as abnormal meibum quality and ductal dilatation [10,16]. Age-related patients present exhibit a degenerative atrophic pattern of MGD, characterized by acinar loss, ductal shortening, and reduced meibum-secreting cells. The meibomian gland dropout rate increases significantly with age (approximately 15% per decade), with more severe inferior eyelid gland damage than superior eyelids. Even after ductal unblocking, secretory function cannot be fully restored [17,18], and 3D meibography shows 73% of age-related patients exhibit an acinar atrophy phenotype [19].

3. Corneal Nerve and Tear Function

Confocal microscopy studies confirm that corneal nerve fiber density (CNFD) in healthy Chinese adults is 15.8 ± 5.2 mm/mm^2^ in individuals younger than 65 years and 14.4 ± 4.3 mm/mm^2^ in those aged 65 years and older. Corneal nerve branch density (CNBD) is also significantly higher in the younger group than in the age-related group, consistent with better corneal nerve sensitivity in younger individuals [20]. The age-related group exhibits impaired peripheral nerve myelin integrity and axonal structure, with a high prevalence of demyelination (approximately 65–70%) and axonal loss, leading to a 25–35% reduction in nerve conduction velocity [21].

Tear function assessment shows Schirmer I test values of 11.3 ± 2.7 mm/5 min in the presenile group, compared with 6.8 ± 2.2 mm/5 min in the age-related group, consistent with the age-associated decline in basal tear secretion [22,23]. The age-related group exhibits a 34.7% reduction in tear film muco-aqueous layer thickness compared with young and middle-aged adults, which significantly limits the efficacy of aqueous tear supplementation alone [24].

4. Characteristics of High-Risk Subgroups

- Presenile Cataract High-Risk Subgroups:

Metabolically associated subgroup: In the presenile cataract population aged 40–55 years, approximately 30–40% present with metabolically associated cataract (e.g., hyperhomocysteinemia, dyslipidemia, or diabetes mellitus). Compared with non-metabolic presenile cataract patients, these individuals exhibit a significantly thinner tear film lipid layer (by approximately 30–40%) preoperatively, and their postoperative dry eye symptoms persist for an additional 2–3 months due to impaired meibomian gland function and delayed ocular surface recovery [25,26,27]. Female subgroup: Female patients experience approximately 30% lower meibomian gland lipid secretion compared with age-matched males due to fluctuations in sex hormones (notably decreased estrogen levels), resulting in impaired tear film stability and a longer duration of postoperative dry eye [9,28]. Comorbidity subgroup: Diabetic patients with concurrent rosacea have a significantly higher risk of dry eye disease (SHR: 1.55, 95% CI: 1.38–1.75), suggesting that this comorbidity profile may influence postoperative inflammatory recovery [29]. Psychologically associated subgroup: 18% have preoperative anxiety (anxiety scale score ≥ 50), predisposing them to postoperative pain-associated dry eye symptoms (pain score ≥ 4) [30].

- Age-Related Cataract High-Risk Subgroups:

Nerve demyelination subgroup: Corneal nerve degeneration consistent with demyelinating changes is highly prevalent in age-related cataract patients. This subgroup demonstrates significantly delayed recovery of corneal sensitivity postoperatively, with functional restoration typically requiring 3–6 months [2,31]. Mucin deficiency subgroup: Age-related patients commonly present with reduced tear film mucin layer thickness, which falls below normal physiological thresholds in a large proportion of cases, requiring targeted mucin-promoting topical agents [32]. Meibomian gland fibrosis subgroup: In addition to glandular atrophy, age-related patients with MGD frequently exhibit glandular fibrosis, characterized by approximately 45% higher collagen content, which further reduces ductal elasticity and secretory function [33].

#### 3.1.2. Surgical Trauma Response and Recovery Trajectory

1. Postoperative Dry Eye Severity

Mechanical stimulation, phacoemulsification-induced thermal injury, corneal nerve transection via clear corneal incisions, and the cytotoxicity of preservatives in postoperative eye drops can all disrupt ocular surface homeostasis [34]. Presenile cataract patients exhibit more pronounced postoperative dry eye symptoms: due to high visual demands in work and study, they are more sensitive to dryness, foreign body sensation, and visual fatigue, with a significantly higher 1-month postoperative OSDI score (35–45 points) compared with the age-related group (25–35 points) [35]. Preoperative anxiety has been identified as an independent risk factor for postoperative dry eye in cataract patients. Elevated preoperative anxiety levels are significantly associated with increased subjective dry eye symptoms, reduced tear film stability, and decreased tear secretion, ultimately leading to a higher incidence and severity of postoperative dry eye [36].

2. Differences in Recovery Trajectories

Postoperative dry eye in age-related cataract patients typically resolves within 1–3 months, while presenile patients experience prolonged dry eye (3–6 months) due to suppressed corneal nerve repair from high visual load and reduced ocular surface compensatory capacity, with some progressing to chronic dry eye [37]. A meta-analysis published in BMC Ophthalmology (2025) [38] including 20 studies and 1694 eyes demonstrated that tear break-up time (TBUT) remains significantly reduced at 3 months after cataract surgery. Based on indirect comparisons from existing cohort studies, presenile cataract patients may experience longer postoperative dry eye duration (3–6 months), whereas age-related patients mostly recover within 1–3 months; however, this difference requires validation through direct head-to-head studies. Postoperative corneal nerve demyelination is more pronounced in the age-related group, delaying sensitivity recovery [31].

3. Tolerance to Refractive IOLs

Presenile cataract patients exhibit lower tolerance to premium IOLs, such as multifocal and extended-depth-of-focus lenses. These IOLs require stable tear film; presenile patients with dry eye are more prone to postoperative adverse visual phenomena, including glare, halos, and decreased contrast sensitivity [23]. Objective visual quality analysis using OQAS demonstrates that poor tear film stability directly increases the objective scattering index (OSI) and decreases the modulation transfer function (MTF), with these effects being more pronounced in the presenile group [24] (see Table 1).

### 3.2. Mechanisms of Perioperative Dry Eye

#### 3.2.1. Corneal Nerve Injury and Repair Mechanisms

Corneal nerves are essential for maintaining tear film stability. Clear corneal incisions in cataract surgery inevitably transect corneal nerve fibers, reducing corneal sensitivity and reflex tear secretion [39]. Although presenile patients have higher corneal nerve density than age-related patients, their nerves have richer branches and intact myelin, which may contribute to more pronounced responses to surgical trauma [20,40,41]. Additionally, presenile patients resume high visual load shortly after surgery, and persistent visual fatigue may further inhibit the expression of nerve growth factors (e.g., GAP-43), potentially prolonging dry eye duration [42].

Age-related cataract patients exhibit significantly impaired corneal nerve regeneration: age-related downregulation of neurotrophic factors (e.g., NGF, BDNF) and reduced Schwann cell function delay nerve repair [43]. Moreover, the age-related group exhibits severe corneal nerve myelin damage (approximately 68% demyelination), obstructing nerve signal conduction; even if nerve fibers regenerate, functional recovery remains delayed [31]. However, age-related patients have lower postoperative visual demands, which may minimize the “functional impact” of nerve injury, potentially resulting in milder subjective symptoms than in the presenile group [44].

#### 3.2.2. Age-Specific Inflammatory Characteristics

The ocular surface of age-related cataract patients is characterized by chronic low-grade inflammation (senescence-associated secretory phenotype, SASP). Senescent cells continuously secrete pro-inflammatory cytokines such as IL-1β, IL-6, and TNF-α, forming a persistent microinflammatory environment [44]. Surgical trauma superimposed on this background may induce a mild but sustained inflammatory response, potentially contributing to prolonged postoperative dry eye [45].

In contrast, presenile cataract patients exhibit predominantly acute ocular surface inflammation. A study in Scientific Reports demonstrated a sharp increase in IL-1β and IL-6 on postoperative day 1, with IL-1β remaining above baseline at 60 days [46]. Analyses indicate that HIF-1α and IRF4 positively correlate with postoperative dry eye risk, whereas SFRP-5 acts as a protective factor [47]. This acute inflammatory pattern may be consistent with the “early peak and rapid resolution” of subjective symptoms. Presenile patients with diabetes complicated by rosacea experience more severe postoperative inflammation [29].

#### 3.2.3. Two Core Patterns of Meibomian Gland Dysfunction

MGD differs fundamentally between the two populations.

- Presenile group—”pump failure” pattern:

Reduced blink rate due to prolonged VDT use impairs the meibomian gland “pumping” mechanism, causing lipid stasis in ducts. Acinar secretory function and ductal structure remain intact, and physical unblocking rapidly restores function [15]. Female presenile patients have approximately 30% reduced meibum synthase activity due to decreased estrogen, exacerbating lipid stasis [28].

- Age-related group—”gland exhaustion + fibrosis” pattern:

Age-related acinar apoptosis, ductal atrophy, and reduced meibum-secreting cells combine with glandular fibrosis (approximately 45% increased collagen content) [33]. Even after ductal unblocking, secretory function is not fully restored, and these patients respond poorly to physical therapy, requiring lipid replacement therapy [17,48].

#### 3.2.4. Common Oxidative Stress Pathway and Age Differences

Presenile cataract and dry eye share a common oxidative stress mechanism: oxidative products from lens aging diffuse to the ocular surface, accelerating corneal epithelial cell apoptosis [49]. However, the source and magnitude of oxidative stress differ.

- Presenile group: These patients are mainly exposed to exogenous factors (ultraviolet radiation, blue light damage, smoking, and VDT syndrome), Systemic oxidative stress markers (malondialdehyde and protein carbonyls) are significantly elevated in presenile cataract patients compared with age-matched healthy controls, as measured in blood samples [50]. Approximately 35% of presenile patients have metabolically related cataracts with even higher oxidative stress levels [51,52].
- Age-related group: This group is primarily affected by endogenous aging (mitochondrial dysfunction, and approximately 50% reduced activity of antioxidant enzymes such as SOD, CAT, and GSH-Px), impairing ROS clearance [53].

#### 3.2.5. Other Mechanistic Differences

Presenile patients with preoperative anxiety are prone to “postoperative pain-related dry eye,” which may be associated with anxiety-mediated increased nerve sensitivity and inflammatory factor release [30]. The age-related group exhibits significant tear film mucin layer deficiency, related to reduced mucin-secreting cell function, decreasing tear film adhesion and exacerbating dry eye [32] (see Table 2).

## 4. Discussion

### 4.1. Age-Stratified Clinical Management Implications

**Figure 2:**
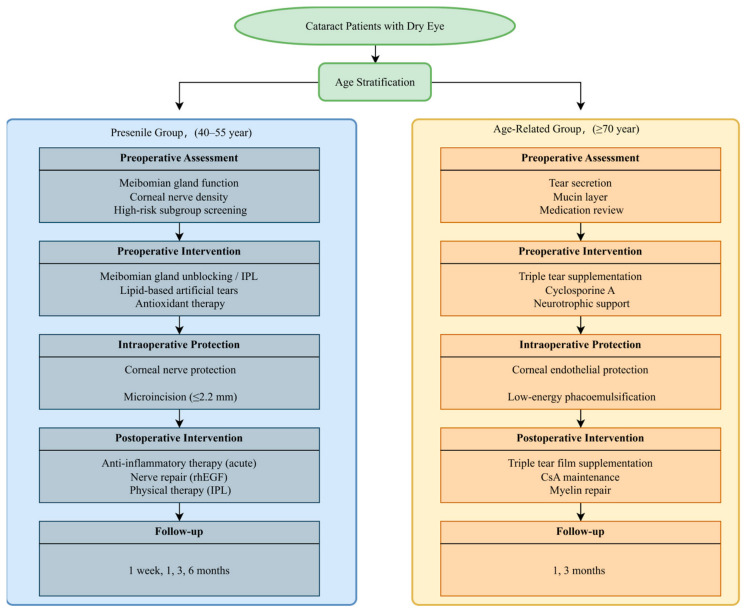
The algorithm shows the key differences between presenile cataract (40–55 years, left column) and age-related cataract (≥70 years, right column) across five management phases: preoperative assessment, preoperative intervention, intraoperative protection, postoperative intervention, and follow-up. Abbreviations: IPL, intense pulsed light; rhEGF, recombinant human epidermal growth factor; CsA, cyclosporine A.

Figure 2 Age-stratified management algorithm for perioperative dry eye in presenile and age-related cataract patients.

Based on the differences discussed above, perioperative management should be age-stratified and subgroup-targeted. Detailed protocols (drug names, concentrations, dosages, and treatment duration) are presented in Table 3.

#### 4.1.1. Key Preoperative Assessment

1. Presenile Cataract Patients

Basic assessment involves meibomian gland function (3D meibography (Keratograph 5M, Oculus, Wetzlar, Germany), meibum quality scoring, MGYLS measurement), corneal nerve status (confocal microscopy (Heidelberg Retinal Tomograph III with Rostock Corneal Module, Heidelberg Engineering, Heidelberg, Germany) for density and branching patterns), and tear film stability (TBUT and tear film interferometry (LipiView, TearScience, Morrisville, NC, USA) [54,55,56,57]. Subgroup screening involves metabolic indicators (homocysteine, blood lipids), sex hormone levels (females), glycated hemoglobin (in diabetic patients), and anxiety scale scoring [28,29,30,51,52]. Premium IOL candidate assessment involves rigorous ocular surface risk stratification using tear film interferometry to evaluate breakage patterns; patients with multifocal tear film break-up should be selected with caution [23,24,57].

2. Age-Related Cataract Patients

Basic assessment involves evaluation of tear secretion function (Schirmer I test (Schirmer test strips, Tianjin Jingming New Technological Development Co., Ltd., Tianjin, China), tear meniscus height), meibomian gland degeneration (dropout rate, fibrosis degree), and corneal nerve myelin integrity (confocal microscopy) [6,58,59]. Subgroup screening involves tear film mucin layer thickness, systemic comorbidities (e.g., diabetes, Sjögren’s syndrome) [7,32]. Comorbidity assessment includes a review of systemic medications affecting tear production (e.g., antihypertensives, antidepressants) [7,45].

#### 4.1.2. Preoperative Intervention Strategies

1. Presenile Cataract Patients (2–4 weeks preoperatively)

- Core goals:

Relieve meibomian gland obstruction, mitigate oxidative stress, enhance corneal nerve sensitivity, and address high-risk subgroups [60].

- Meibomian gland interventions:

Professional expression: Regular meibomian gland expression (four sessions preoperatively) [61]. Targeted therapy involves ductal probing for refractory cases and intense pulsed light (IPL) for severe MGD [11,54,62].

- Pharmacological intervention:

Artificial tears: Preferential use of lipid-containing formulations [63]. Antioxidant therapy: Topical vitamin C combined with oral antioxidants, supplemental folate, and B vitamins for metabolically at-risk subgroups [50,64]. Subgroup-specific intervention: Hormonal regulation for female patients (investigational); anti-inflammatory therapy for diabetes with rosacea; and psychological support for patients with high anxiety scores (off-label use for dry eye prevention) [28,29,30].

- Behavioral intervention:

Blinking training, implementation of the 20-20-20 rule (focusing on an object 20 feet away for 20 s every 20 min), limiting daily screen time to ≤4 h [65].

2. Age-Related Cataract Patients (2–4 weeks preoperatively)

- Core goals: Enhance tear secretion and mucin layer function, stabilize the tear film, and preserve corneal nerve myelin [66].
- Pharmacological intervention:

Artificial tears: Preservative-free formulations, including hyaluronic acid and mucin-promoting agents [54,63,67]. Anti-inflammatory therapy: topical cyclosporine A (Zirun®, Shenyang Xingqi Eye Pharmaceutical Co., Ltd., Shenyang, China) (initiated 4 weeks preoperatively) and anti-fibrotic agents for meibomian gland fibrosis [55,68]. Neuroprotection: Topical neurotrophic factors (investigational, region-specific) [31]. Nutritional support: Omega-3 fatty acids and vitamin A supplementation [69].

- Physical therapy:

Warm compresses and gentle lid margin massage [17,70].

For detailed protocols (drug names, concentrations, dosages) and evidence classification (standard care vs. investigational/off-label), see Table 3.

#### 4.1.3. Intraoperative Protection Strategies

Both groups adhere to minimally invasive surgical principles (microincision, low phacoemulsification energy, short effective emulsification time, avoiding excessive eyelid speculum compression), with group-specific priorities [71].

- Presenile group:

Corneal nerve protection: microincision approach, low phacoemulsification energy, and shortened effective emulsification time [72].

- Age-related group:

Corneal endothelium and nerve myelin protection: controlled phacoemulsification energy and use of viscoelastic agents [73,74].

#### 4.1.4. Postoperative Intervention Protocol

1. Presenile Cataract Patients (1–3 months postoperatively)

- Anti-inflammatory therapy:

Topical corticosteroids with gradual tapering combined with NSAIDs [75]. Maintenance phase: Low-potency corticosteroids for up to 6 weeks.

Nerve repair: Topical neurotrophic factors combined with oral neuroprotective agents [76].

Physical therapy: Resumption of meibomian gland unblocking 1 month postoperatively and targeted IPL therapy for selected patients [57,62].

- Subgroup management:

Metabolically at-risk patients should continue oral folic acid and vitamin B6 for 3 months [51,52]. Patients with high anxiety scores should continue psychological intervention, and ibuprofen (200 mg twice daily) for pain scores ≥ 4 [30].

Follow-up frequency: 1 week: TBUT + corneal fluorescein staining (CFS); 1 month: corneal confocal microscopy; 3 months: MGD indicators + tear film interferometry; 6 months: (dry eye symptom assessment [77].

2. Age-Related Cataract Patients (1–3 months postoperatively)

- Repair therapy:

Tear film supplementation: Triple therapy targeting aqueous, mucin, and lipid layers [57,67]. Corneal nerve myelin repair: Topical neurotrophic factors combined with oral neuroprotective agents [31]. Anti-inflammatory maintenance: Long-term cyclosporine A for chronic inflammation control [78].

- Subgroup management:

Mucin-deficient patients should continue mucin-promoting therapy [68].

Follow-up frequency should involve anti-fibrotic therapy with warm compresses [80].

### 4.2. Research Limitations and Future Directions

#### 4.2.1. Limitations of Existing Research

1. Absence of head-to-head comparative studies: (core limitation). To date, no prospective study has directly compared perioperative dry eye between presenile and age-related cataract populations. Consequently, all comparisons in this review are based on indirect evidence synthesis rather than direct comparisons. This approach introduces potential sources of bias and confounding, including population heterogeneity (differences in the prevalence of high myopia, diabetes, and other confounders), study design variability (cross-sectional, cohort, case-control), measurement inconsistency (different methods for tear film assessment), and publication bias. Therefore, the conclusions of this review should be considered hypothesis-generating rather than definitive. Future prospective cohort studies with strict age stratification and matching for confounding factors are urgently needed [81].
2. Inconsistent age definition criteria: The age range defining for presenile cataract lacks consensus across studies (lower limit 35–40 years; upper limit 50–55 years), limiting direct comparability and generalizability of findings [79].
3. Insufficient subgroup research: There is a scarcity of specialized intervention studies for high-risk presenile subgroups (metabolically related, female anxious patients) and age-related subgroups (nerve demyelination, mucin deficiency) [28,29,30,32,68].
4. Limited evidence for emerging technologies: Clinical validation of novel diagnostic and therapeutic modalities, such as 3D meibography, tear film interferometry, and targeted IPL, remains insufficient, with a lack of multicenter randomized controlled trials [57,62].
5. Exclusion of transitional age range: Patients aged 55–70 years were intentionally excluded from our analytical framework to maximize contrast between the presenile (40–55 years) and age-related (≥70 years) groups. Consequently, findings may not be directly applicable to this transitional population, who may exhibit with mixed or intermediate dry eye characteristics. Future studies specifically addressing patients aged 55–70 years are needed to determine whether they align more closely with the presenile or age-related cataract group, or whether a distinct management strategy is required.

#### 4.2.2. Future Research Directions

1. Age subgroup-stratified management guidelines and standardized clinical pathways for different age groups and subgroups should be established based on existing evidence to harmonize assessment and intervention protocols [82].
2. Individualized risk prediction models integrate age, subgroup characteristics, and preoperative ocular surface indicators should be constructed to predict individual postoperative dry eye risk and prognosis [83].
3. Targeted therapeutic agents should be investigated and mitochondria-targeted antioxidants (e.g., MitoQ) developed for oxidative stress in the presenile group, alongside neurotrophic-targeted drugs for myelin damage in the age-related group [31,50].
4. Multicenter studies on emerging technologies should be carried out. The long-term efficacy of individualized interventions guided by 3D meibography and targeted IPL should be validated [57,62].
5. AI-assisted clinical decision systems should be established. We should develop artificial intelligence systems integrating age, etiology, and ocular surface characteristics to achieve automated, precise matching of assessment, diagnosis, and treatment plans [84].

## 5. Conclusions

Perioperative dry eye exhibits inherent differences between presenile and age-related cataract patients: the presenile group is dominated affected by evaporative dry eye, characterized by meibomian gland functional overload, high-sensitivity corneal nerve injury, pronounced postoperative acute inflammation, and prolonged recovery. High-risk subgroups include metabolically at-risk, female, and high-anxiety patients. The age-related group is mainly mixed dry eye, characterized by meibomian gland degenerative atrophy, dual tear secretion and mucin deficiency, chronic inflammatory background, disrupted corneal nerve myelin integrity, and impaired repair capacity. Clinical management should adopt age-stratified combined with subgroup-targeted management strategies: the presenile group requires intensified preoperative targeted MGD intervention, intraoperative nerve protection, and postoperative acute inflammation control, with additional metabolic regulation and psychological support for special subgroups. The age-related group should focus on triple-layer tear film supplementation, postoperative corneal nerve myelin repair, and chronic inflammation management. Precise management can effectively reduce postoperative dry eye incidence, enhance surgical outcomes, and improve both visual quality and overall quality of life across different age groups of cataract patients.

## Figures and Tables

**Table 1:** Comparative clinical characteristics of perioperative dry eye in presenile and age-related cataract patients.

Clinical Characteristic	Presenile Cataract Group (40–55 Years)	Senile Cataract Group (≥70 Years)	Evidence Source
Preoperative dry eye prevalence	50–65%	30–45%	[5,6]
Main dry eye type	Predominantly evaporative dry eye	Mixed dry eye (evaporative + aqueous deficiency + mucin deficiency)	[7,8]
Meibomian gland function	Functional obstruction with ductal dilatation, intact glandular structure	Degenerative acinar atrophy, more severe inferior eyelid impairment	[10,11,12,13,14]
Tear secretion (Schirmer I, mm/5 min)	10–16, mildly decreased or normal	4–9, moderate to severe reduction	[17,18]
Corneal nerve status	Moderate density, rich branches, low demyelination rate (<15%)	Significantly reduced density, extensive myelin loss, slowed conduction	[15,16]
Preoperative MGD prevalence	55–70%	50–63%	[10,11,29]
1-month postoperative OSDI score	33–43 points, moderate-severe discomfort	23–33 points, mild-moderate discomfort	[31]
3-month postoperative TBUT recovery	Recovered to 70–80% of baseline, slow recovery	Recovered to ≥90% of baseline, rapid recovery	[34]
Dry eye duration	3–6 months, partial progression to chronic condition	1–3 months, mostly spontaneous remission	[33,34]
Tear film breakage pattern	Multifocal breakage	Global breakage	[18,19]
Multifocal IOL tolerance	Poor, high incidence of glare and halos	Fairly good, less photic complaints	[18]
High-risk subgroups	Metabolic disorders, female, diabetes, rosacea, preoperative anxiety	Corneal nerve demyelination, mucin deficiency, meibomian gland fibrosis	[20,21,22,23,24,25,26,27,28]
Subjective symptom sensitivity	High, notable impact on daily life and work	Moderate, better symptom tolerance	[31]

Note: MGD = meibomian gland dysfunction; TBUT = tear film break-up time; OSDI = Ocular Surface Disease Index; IOL = intraocular lens.

**Table 2:** Differential mechanisms of perioperative dry eye in presenile and age-related cataract patients.

Mechanistic Aspect	Presenile Cataract Group (40–55 Years)	Senile Cataract Group (≥70 Years)	Evidence Source
Corneal nerve injury mechanism	High-sensitivity injury: rich branches, intact myelin, intense surgical trauma response; high visual load inhibits GAP-43 expression, delaying repair	Impaired regeneration + myelin damage: downregulated neurotrophic factors (NGF, BDNF); 68% demyelination rate, reduced conduction velocity	[15,16,36,37,38,39,40]
Inflammatory characteristics	Acute inflammation-dominant: sharp postoperative increase in IL-1β/IL-6, resolution within 1–2 weeks; more severe inflammation in comorbid subgroups	Chronic low-grade inflammation: SASP-related persistent low-level elevation of IL-1β/TNF-α, prolonged inflammation	[41,42,43,44]
Meibomian gland dysfunction pattern	“Pump failure”: lipid stasis due to reduced blink rate, intact glandular structure; hormone-mediated meibum synthesis reduction in females	“Gland exhaustion + fibrosis”: acinar apoptosis/ductal atrophy; 45% increased collagen content, decreased ductal elasticity	[9,24,29,45,46]
Oxidative stress source/intensity	Exogenous-dominant (UV, blue light, smoking, metabolic abnormalities); 2.7-fold higher marker levels, 35% increased corneal epithelial apoptosis	Endogenous-dominant (mitochondrial dysfunction, 50% reduced antioxidant enzyme activity)	[48,49,50,51,52]
Tear film instability mechanism	Lipid layer deficiency (abnormal meibum quality), multifocal tear film breakage	Triple deficiency (lipid + aqueous + mucin), global tear film breakage	[18,19]
Repair capacity	High nerve repair potential, inhibited by visual load and anxiety	Low repair potential, but low postoperative visual load	[40]
Molecular marker characteristics	Elevated HIF-1α/IRF4 (positive correlation with dry eye risk); inhibited GAP-43 expression	Persistent low-level expression of SASP-related factors (IL-1β/TNF-α); decreased neurotrophic factor levels	[38,44]

Note: IL = interleukin; TNF = tumor necrosis factor; NGF = nerve growth factor; BDNF = brain-derived neurotrophic factor; SASP = senescence-associated secretory phenotype; GAP-43 = growth-associated protein 43.

**Table 3:** Comparative perioperative management strategies for dry eye in presenile and age-related cataract patients.

Management Phase	Presenile Cataract Group (40–55 Years)	Senile Cataract Group (≥70 Years)	Evidence Source
Key preoperative assessment	3D meibography + corneal nerve branch density + tear film interferometry; metabolic/hormone/anxiety screening	Schirmer I + tear film mucin layer thickness + nerve myelin integrity; systemic medication review	[53,54,55,56,57,58,59,60]
Core preoperative intervention	Obstruction relief + oxidative stress control: meibomian gland unblocking/IPL, lipid-supplementing artificial tears, antioxidants	Triple supplementation + neuroprotection: aqueous/mucin/lipid supplements, cyclosporine A, mouse nerve growth factor	[61,62,63,64,65,66,67,68,69,70,71,72,73]
Preoperative intervention duration	2–4 weeks (rapid physical therapy efficacy)	4 weeks (sufficient cyclosporine A efficacy)	[54,62]
Intraoperative protection priority	Corneal nerve protection (≤2.2 mm microincision, low-energy phacoemulsification)	Corneal endothelium + myelin protection (energy/time control, viscoelastic agent protection)	[74,75,76,77]
Postoperative anti-inflammatory strategy	High-dose steroids (acute phase intensification) + NSAIDs	Cyclosporine A (long-term maintenance) + low-concentration steroids	[78,79]
Postoperative repair focus	Nerve regeneration (rhEGF + methylcobalamin) + targeted IPL	Triple tear film supplementation + myelin repair (mouse nerve growth factor)	[27,56,64,70,80]
Artificial tear preference	Lipid-supplementing formulations	Aqueous + mucin-promoting formulations	[65]
Subgroup-specific intervention	Females: estrogen gel; metabolic abnormalities: folic acid + vitamin B6; anxiety: psychological intervention	Mucin deficiency: high-concentration diquafosol; fibrosis: pirfenidone	[24,25,26,28,50,51,71]
Follow-up frequency	1 week, 1 month, 3 months, 6 months	1 month, 3 months	[81,82]

Note: IPL = intense pulsed light; rhEGF = recombinant human epidermal growth factor; NSAIDs = non-steroidal anti-inflammatory drugs. Evidence level refers to the Oxford Centre for Evidence-Based Medicine classification; Standard care (guideline-supported, regulatory approved for dry eye): preservative-free artificial tears, cyclosporine A, corticosteroids, diquafosol, IPL. Investigational/off-label (preliminary evidence, not approved for dry eye by major regulatory agencies): estrogen gel, pirfenidone eye drops, oral sertraline. Region-specific/investigational: mouse nerve growth factor (mNGF) eye drops (mainly reported in China).

## Data Availability

No new data were created or analyzed in this study. Data sharing is not applicable to this article.
